# Expanding the mutational spectrum of congenital microcephaly in Pakistani families

**DOI:** 10.3389/fgene.2025.1709083

**Published:** 2026-01-05

**Authors:** Sundas Farooq, Maria Asif, Ansar A. Abbasi, Zahid Latif, Bonsu Ku, Ehtisham Ul Haq Makhdoom, Madiha Shadab, Muzammil Ahmad Khan, Muhammad Muzammal, Raja Waqar, Rameez Nisar, Falak Sher Khan, Sanwal Aslam, Michal R. Schweiger, Muhammad Sajid Hussain

**Affiliations:** 1 Department of Zoology, Mirpur University of Science and Technology (MUST), Mirpur, Pakistan; 2 Cologne Center for Genomics (CCG), University of Cologne, Faculty of Medicine and University Hospital Cologne, Cologne, Germany; 3 Cologne Excellence Cluster on Cellular Stress Responses in Aging-Associated Diseases (CECAD), University of Cologne, Cologne, Germany; 4 Department of Zoology, University of Azad Jammu and Kashmir, Muzaffarabad, Pakistan; 5 Orphan Disease Therapeutic Target Research Center, Korea Research Institute of Bioscience and Biotechnology, Daejeon, Republic of Korea; 6 Neurochemicalbiology and Genetics Laboratory (NGL), Department of Physiology, Faculty of Life Sciences, Government College University, Faisalabad, Pakistan; 7 Gomal Center of Biochemistry and Biotechnology, Gomal University, Dera Ismail Khan, Khyber-Pakhtunkhwa, Pakistan; 8 Department of Zoology, University of Kotli, Kotli, Pakistan; 9 Department of Biological Sciences, University of Sialkot, Sialkot, Pakistan; 10 School of the Environment and Safety Engineering, Jiangsu University, Zhenjiang, China

**Keywords:** primary microcephaly, ASPM, WDR62, CPAP, whole exome sequencing, TCP10 domain

## Abstract

Autosomal recessive primary microcephaly (MCPH) is a genetically heterogeneous neurodevelopmental disorder characterized by a markedly reduced head circumference (−3 to −5 standard deviations) at birth, with relatively preserved brain architecture. Affected individuals often present with mild to moderate intellectual disability, and the condition is more prevalent in populations with high rates of consanguinity, such as Pakistan. To date, pathogenic variants in at least 32 genes have been associated with MCPH, with *ASPM* and *WDR62* accounting for the majority of cases (68% and 14%, respectively). In this study, we investigated four consanguineous families with congenital microcephaly and identified three novel variants in *CPAP, WDR62*, and *ASPM*. In Family 1, we identified a novel missense variant (c.3947C>A; p. (Thr1316Lys) in *CPAP* (NM_018451.4) located within the highly conserved TCP domain, which mediates interactions with other MCPH proteins, including STIL and CEP135. Family 2 harbored a previously unreported splice-site variant, c.2867 + 5G>T, in *WDR62* (NM_001083961.2). In Families 3 and 4, we identified one novel (c.3188T>G; p. (Leu1063*)) and one previously reported (c.9730C>T; p. (Arg3244*)) pathogenic variant in *ASPM* (NM_018136.4). Computational analyses and structural modeling indicated that all these variants are likely deleterious, disrupting normal protein function. Our findings expand the mutational spectrum of *CPAP* and *WDR62* and reinforce *ASPM* as the most frequently mutated gene underlying MCPH in the Pakistani population.

## 1 Introduction

Autosomal recessive primary microcephaly (MCPH [MIM 251200]) is a rare neurodevelopmental disorder of the brain characterized by reduced head circumference (−3 to −5 standard deviations [SD]) and variable degrees of intellectual disability and cognitive impairment. It is genetically and clinically heterogeneous and occurs most frequently in populations where consanguineous marriages are common (Khan et al., 2022; Létard et al., 2018). Although MCPH is rare worldwide, its incidence is considerably higher in countries such as Pakistan (1 in 10,000) compared with Europe, where it occurs sporadically (1 in 1,000,000) (Asif et al., 2023). The high prevalence in Pakistan is attributed to frequent consanguineous marriages and a lack of genetic counseling, which increases the risk of recessive disorders.

To date, pathogenic variants in at least 32 genes have been identified as the underlying cause of MCPH, highlighting its genetic heterogeneity (Waseem et al., 2021; Carvalhal et al., 2022). Among these, *ASPM* is the most frequently mutated gene, accounting for approximately 68% of reported cases, followed by *WDR62* (14%) and *MCPH1* (8%) (Létard et al., 2018; Zaqout et al., 2017). Most causative proteins are localized at the centrosome and are involved in neurogenic mitosis, explaining the neuropathological features of MCPH (Asif et al., 2023). The *ASPM* gene spans 62 kb, contains 28 exons, and plays a critical role in spindle function. The *WDR62* gene, located on chromosome 19q13.12, comprises 32 exons with a genomic length of 502,309 bp. Variants in *WDR62* have been associated with primary microcephaly, intellectual disability, and cerebral hypoplasia (Nicholas et al., 2010).

Another gene implicated in MCPH is *CPAP* (previously known as *CENPJ*), which encodes a centrosomal protein of 1,338 amino acids. It contains several functional domains, including a 112–amino acid microtubule-disrupting motif (PN2-3), a 149–amino acid coiled-coil domain, and a TCP (T-complex protein 10) domain at the C-terminus (Zheng et al., 2014). According to HGMD (2025.1), 47 mutations in *CPAP* have been reported, the majority in Pakistani families. Notably, five Pakistani families were found to carry a recurrent frameshift variant, c.18delC; p. (Ser7Profs*2), of *CPAP* and proposed as a founder mutation in this population (Bond et al., 2005; Rasool et al., 2020; Sajid et al., 2013).

Proteins associated with primary microcephaly (MCPH) are ubiquitously expressed and play critical roles in the regulation of cell-cycle progression. They localize to essential mitotic structures, including the centrosome, spindle poles, spindle microtubules, kinetochores, cleavage furrow, midbody, and are also components of chromatin-remodeling complexes and the pre-mRNA spliceosome (Asif et al., 2023). *WDR62* encodes a WD repeat–containing protein that localizes to spindle poles during mitosis and is highly expressed in neural progenitor cells and post-mitotic neurons (Yu et al., 2010). Loss of *Wdr62* in murine models results in microcephaly due to depletion of neural progenitor cells, arising from spindle defects and mitotic arrest caused by impaired interaction with Aurora A, ultimately triggering apoptosis of progenitor cells (Chen et al., 2014). ASPM (abnormal spindle-like microcephaly-associated protein), which also localizes to mitotic spindle poles in neuroepithelial cells and is essential for proper cleavage plane orientation, which ensures symmetric, proliferative divisions. Loss or reduction of ASPM disrupts this orientation, diminishing the proliferative capacity of neuroepithelial cells and leading to diminished brain size (Fish et al., 2006). Likewise, perturbations in CPAP, a centrosomal protein required for centriole biogenesis, induce premature differentiation of neuronal progenitors due to delayed cell-cycle entry, a consequence of impaired primary-cilium disassembly (Gabriel et al., 2016).

In the present study, we investigated two families with known and novel variants in the *ASPM* gene and additionally identified two novel variants, one in *WDR62* and the other in *CPAP*. In silico analysis, combined with protein modeling, was employed to further assess the pathogenicity of these disease-causing variants.

## 2 Materials and methods

### 2.1 Family recruitment and ethical considerations

This study was conducted on four consanguineous families from different regions of Azad Kashmir, Pakistan. Written informed consent was obtained from all participants prior to sample collection. Peripheral blood samples were collected from affected individuals and their family members. The study was conducted according to the guidelines of the Declaration of Helsinki and approved by the Institutional Review Board (IRB) of Mirpur University of Science and Technology (MUST), Mirpur, Pakistan (Approval No. ORIC/43-46/2024; dated 28-02-2024).

### 2.2 DNA extraction and sequencing

Genomic DNA was extracted from peripheral blood using a commercial kit (Thermo Fisher Scientific) following the manufacturer’s instructions. Whole-exome sequencing (WES) was performed for the affected individuals using the Agilent SureSelect XT HS Human All Exon V6 enrichment kit. Sequencing was carried out on the Illumina NovaSeq 6000 platform with paired-end reads (2 × 100 bp). Sequencing data were analyzed following established protocols (Hussain et al., 2012). Variant interpretation was performed using the VARBANK pipeline (https://varbank.ccg.uni-koeln.de/varbank2/) from the Cologne Center for Genomics (CCG), University of Cologne. We investigated all variants found in both coding and non-coding regions, including intronic, intragenic, extragenic, intragenic regulatory, and extragenic regulatory. Identified variants were further validated and their co-segregation confirmed within families using Sanger sequencing. Oligonucleotide sequences are shown in the Supplementary Table S1.

### 2.3 Protein structure prediction and *in silico* analysis

To assess the pathogenicity of the identified variants, especially the missense and splice-site variants, we employed several *in silico* prediction tools, as listed in Table 2. For splice site variant, we particularly used SpliceAI, Pangolin, NetGene2 Server, MaxEntScan, Human Splicing Finder, and RNA Splicer. Protein sequence alignment was performed using Clustal Omega from EMBL-EBI (https://www.ebi.ac.uk/jdispatcher/msa/clustalo?stype=protein) to assess evolutionary conservation and potential functional impacts of the CPAP variant. The effects of the CPAP missense variant p. (Thr1316Lys) and the ASPM stop-gained variants p. (Arg3244*) and p. (Leu1063*) were assessed at the atomic level to evaluate potential structural and functional consequences. As the experimental crystal structures of these proteins are not available, three-dimensional models were generated using AlphaFold 3 (https://alphafoldserver.com/).

## 3 Results

### 3.1 Clinical manifestation

**FIGURE 1:**
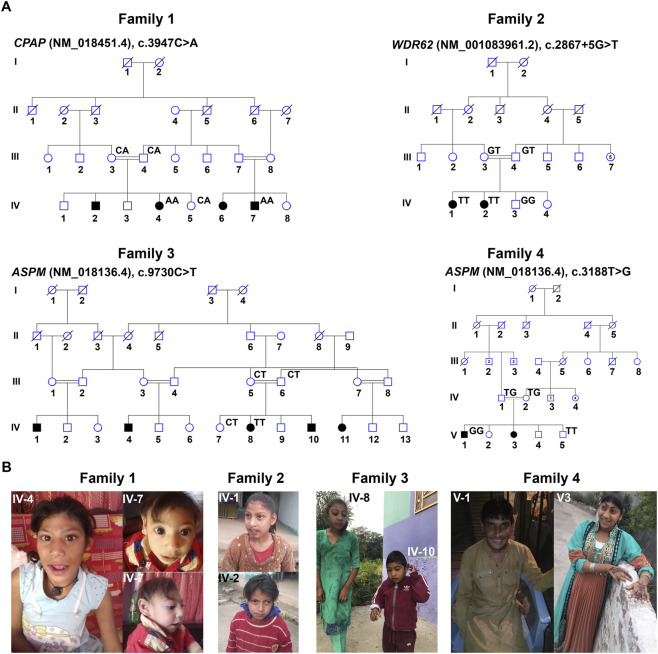
Clinical manifestations associated with pathogenic variants in *CPAP, WDR62*, and *ASPM* in Pakistani families **(A)** Pedigrees of the four Pakistani families investigated in this study. Squares represent males, whereas circles represent females. Black-filled circles and squares indicate affected individuals. A line connecting a male and a female represents a marriage, and double lines indicate a consanguineous marriage. A diagonal line through a symbol indicates a deceased individual. Roman numerals denote each generation of the family, whereas the numbers below each symbol represent the individual’s number within that generation. For each tested individual, the corresponding genotype is displayed above their symbol in the pedigree. **(B)** Facial photographs of Pakistani families carrying pathogenic variants in *CPAP*, *WDR62*, and *ASPM*.

We recruited four consanguineous Pakistani families affected by congenital primary microcephaly. All affected individuals were born to clinically asymptomatic parents and shared a homozygous genetic background due to consanguinity (Figure 1A). Clinically, patients exhibited hallmark features of primary microcephaly, including markedly reduced head circumference (−3 to −5 standard deviations [SD]), variable intellectual disability (mild to severe), sloping forehead, protruding ears, and, in some cases, aggressive behavior (Figure 1B; Table 1). None of the affected individuals had received treatment for microcephaly, and thus, no medical intervention history was available.

**TABLE 1:** Clinical and molecular manifestations of individuals carrying variants in *CPAP*, *WDR62*, and *ASPM*.

Family ID	Family 1		Family 2		Family 3		Family 4	
Gene	*CPAP*		*WDR62*		*ASPM*		*ASPM*	
Variant	c.3947C>A; p. (Thr1316Lys)		c.2867 + 5G>T, p. (?)		c.9730C>T; p. (Arg3244*)		c.3188T>G; p. (Leu1063*)	
Zygosity	Homozygous		Homozygous		Homozygous		Homozygous	
Affected individual	IV4	IV-7	IV-1	IV-2	IV-8	IV-10	V-1	V-3
Age (years)	10	6	12	10	8	5	16	15
Sex	Female	Male	Female	Female	Female	Male	Male	Female
HC^!^ (SD)	−6	−10.3	−9.2	−9	−11.8	−11	−8	−10.6
Height (SD)	−2	−3.53	−3	−3	−7.5	−7	−2.2	−4.93
Weight (SD)	3	−2.3	−2.89	−4	−3.1	−1.5	−2.4	−3.06
Body mass index (Kg/m2)	12 (underweight)	14.4 (underweight)	13.43 (underweight)	16.8	15.6	16.8	16.1	19.5
Intellectual disability	Moderate	Moderate	Mild	Moderate	Moderate	Moderate	Severe	Severe
Impaired cognition	+	+	+	+	+	+	+	+
Delayed motor development	-	-	-	-	-	-	-	-
Speech	Normal	Normal	Absent	Absent	Few words	Absent	Normal	Normal
Seizures	-	-	-	-	-	-	-	-
Facial feature	Sloping forehead	Sloping forehead	Slopping forehead	Slopping forehead	Slopping forehead	Slopping forehead	Slopping forehead	Slopping forehead
Behavior	Friendly	Friendly	Aggressive	Friendly	Friendly	Friendly	Aggressive	Aggressive

^!^ HC, refers to head circumference. “+” indicates the presence of the phenotype, whereas “–” indicates its absence.

### 3.2 Pathogenic variants identification

Whole-exome sequencing revealed four disease-associated variants across the families (Supplementary Table S2). In Family 1, we detected a novel missense variant (c.3947C>A; p. (Thr1316Lys)) in *CPAP* (NM_018451.4) located in exon 17 (Figure 1A; Table 1). Family 2 harbored a novel splice-site variant, c.2867 + 5G>T, in exon 23 of the *WDR62* (NM_001083961.2) (Figure 1A; Table 1). Family 3 carried a previously reported nonsense variant (c.9730C>Tc; p. (Arg3244*)) in exon 24 of the *ASPM* (NM_018136.4) (Figure 1A; Table 1). In Family 4, another stop-gained but novel variant (c.3188T>G; p. (Leu1063*)), in exon 13 of *ASPM* (NM_018136.4) was identified (Figure 1A; Table 1). Sanger sequencing confirmed the homozygous state of each variant in affected individuals, whereas available parents were heterozygous carriers (Figure 1A; Supplementary Figure S1). The available unaffected siblings were either heterozygous or homozygous for the wild-type allele, consistent with recessive inheritance (Figure 1A; Supplementary Figure S1). Pathogenicity predictions using multiple *in silico* tools supported the deleterious nature of all four variants (Table 2). According to ACMG criteria, both *ASPM* variants were classified as pathogenic (PVS1, PM2, PP3, PP5), whereas the *WDR62* and *CPAP* variants were classified as variants of uncertain significance (PM2, PP3) (Table 2). Notably, analysis of the WES data from all four families did not identify any additional pathogenic variants that could contribute to the disease phenotype.

**TABLE 2:** Predicted pathogenicity of the variants identified in this study.

Family ID	Family 1	Family 2	Family 3	Family 4
Gene (MIM No.)	*CPAP* (608393)	*WDR62* (604317)	*ASPM* (608716)	*ASPM* (608716)
cDNA variant	NM_018451.5 c.3947C>A	NM_001083961.2 c.2867 + 5G>T	(NM_018136.5) c.9730C>T	(NM_018136.5) c.3188T>G
Protein variant	p. (Thr1316Lys)	p. (?)	p. (Arg3244*)	p. (Leu1063*)
References	Novel	Novel	Known (PMID: 28674240)	Unpublished (RCV000020764.3)^a^
MAF (gnomAD, v4.1.0)	1.86e-6 (3 alleles)	6.20e-7 (1 allele)	4.96e-6 (8 alleles)	Absent
CADD score	29.6	17.17	40.0	36.0
Mutation taster	Disease-causing (78)	Disease-causing	Disease-causing (6)	Disease-causing (6)
PolyPhen-2	Disease-causing (1.00)	−	−	−
REVEL	Disease-causing (0.850)	−	−	−
SIFT	Disease (0.010)	−	−	−
PROVEAN	Deleterious (−5.63)	−	−	−
LRT	Deleterious (0.000000)	−	Deleterious (0.767100)	Deleterious (0.386283)
ACMG classification	VUS (PM2, PP3)	VUS (PM2, PP3)	Pathogenic (PVS1, PM2, PP3, PP5)	Pathogenic (PVS1, PM2, PP3, PP5)
PhD-SNP	Disease (0.859)	−	−	−
SNAP	Disease (0.670)	−	−	−
Meta-SNP	Disease (0.723)	−	−	−
SpliceAI	No effect (0.00)	Donor loss (0.680)	No effect (0.00)	No effect (0.00)
Pangolin	No effect (0.0300)	−0.740	−0.140	−
NetGene2 server	−	Disruption of the wild-type donor site	−	−
MaxEntScan	−	Disruption of the wild-type donor site	−	−
Human splicing finder	−	Alteration of the wild-type donor site	−	−
RNA splicer	−	Exon skipping, frameshift mutation, premature termination	−	−

Abbreviations: MAF, minor allele frequency; CADD, combined annotation dependent depletion; LRT, likelihood ratio test; SIFT, sorting intolerant from tolerant; REVEL, rare exome variant ensemble learner; ACMG, american college of medical genetics and genomics; VUS, variant of uncertain significance; SNAP, Screening for Non-Acceptable Polymorphisms.

^a^ ID, refers to the ClinVar identifier.

**FIGURE 2:**
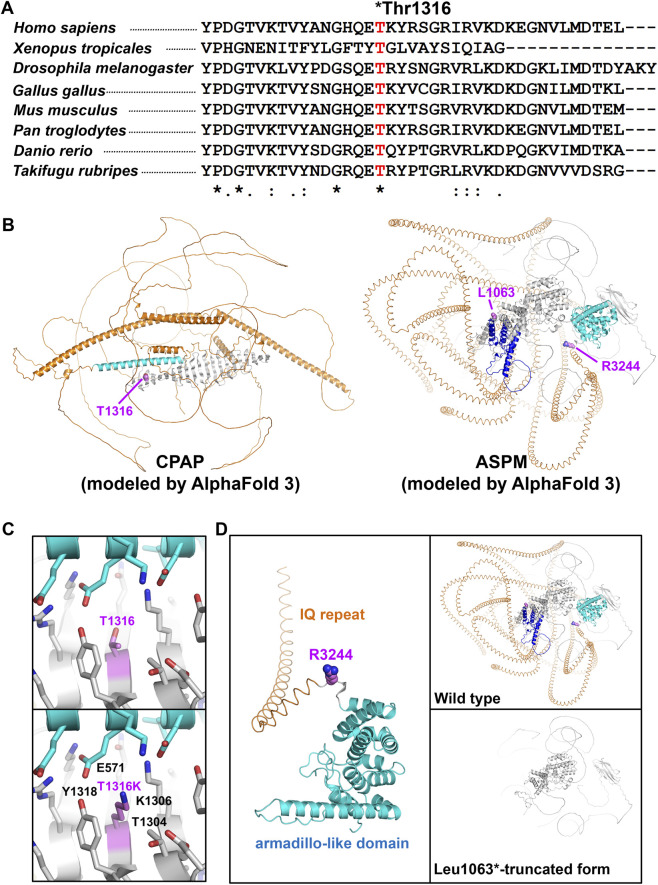
Conservation of the CPAP and molecular modeling of the CPAP and ASPM variants **(A)** Conservation of the CPAP variant among vertebrates. **(B)** Structural models of CPAP (left) and ASPM (right) generated using AlphaFold3. Key amino acids are shown as spheres and labeled. **(C)** Analysis of the T1316K mutation in CPAP. Wild-type (left) and mutant (right) structures highlight steric hindrance at residue 1,316. **(D)** Analysis of ASPM mutations R3244* and L1063*. The R3244* mutation results in loss of the C-terminal armadillo-like domain, whereas the L1063* mutation truncates calponin homology domain 2, IQ repeats, and the armadillo-like domain.

The novel *CPAP* missense variant, p. (Thr1316Lys), is absent from ClinVar and has a high CADD score of 29.6. Although the surrounding region is not conserved among vertebrates, the affected residue is highly conserved (Figure 2A). In gnomAD, it was observed in only three heterozygous alleles among South Asian individuals (allele frequency 1.86e-6), suggesting that it may represent a founder variant.

The *WDR62* splice-site variant is also extremely rare, absent in ClinVar, but observed in gnomAD as a single allele in the non-Finnish European population (allele frequency 6.20e-7). In silico predictions by SpliceAI, Pangolin, NetGene2 Server, MaxEntScan, Human Splicing Finder, and RNA Splice consistently indicated disruption of the canonical donor site, supporting aberrant splicing (Table 2; Supplementary Figure S2).

The *ASPM* nonsense variant p. (Arg3244*) is catalogued in gnomAD with eight total alleles (2 South Asian, 5 non-Finnish European, 1 African/African American). In contrast, the p. (Leu1063*) stop-gain variant was absent from gnomAD but reported in ClinVar, highlighting its rarity in the Pakistani population.

### 3.3 Protein modeling and structural insights

Three-dimensional molecular modeling of CPAP and ASPM variants was performed using AlphaFold 3, as no experimentally determined structures are available for the relevant regions (Figures 2B–D).

For CPAP, the p. (Thr1316Lys) substitution was analyzed at the atomic level. In the AlphaFold 3 structural model, the distal C-terminal region (residues 1,150–1,338) forms a β-sheet that interacts with an α-helix (residues 547–590) (Figure 2B, left). Thr1316 is located at the central α-helix–binding interface of this C-terminal β-sheet. Substitution with lysine, a bulkier residue, is predicted to cause steric hindrance with adjacent residues, including Glu571 in the central α-helix and Thr1304, Lys1306, and Tyr1318 in the β-sheet (Figure 2C). This structural alteration may disrupt binding of proteins that interact with the C-terminus of CPAP.

For ASPM, we assessed the effect of the premature stop codons p. (Arg3244*) and p. (Leu1063*). In the AlphaFold 3 model, the distal C-terminal region (residues 3,249–3,477) forms an armadillo-like domain (Nicholas et al., 2009) (Figure 2B, right; Figure 2D, left). Due to this mutation, if a truncated protein is formed, it lacks the armadillo-like domain.

The p. (Leu1063*) mutation produces a severely truncated ASPM protein lacking not only the C-terminal armadillo-like domain (residues 3,249–3,477) but also calponin homology domain 2 (residues 1,110–1,264) and multiple IQ repeats (residues 1,265–3,242), which are putative actin- and calmodulin-binding modules, respectively (Figure 2D, right) (Kouprina et al., 2005). Therefore, the p. (Arg3244*) and p. (Leu1063*) mutations are likely to disrupt proper protein–protein interactions and subcellular localization of ASPM, resulting in severe loss of function.

## 4 Discussion

In this study, we identified a novel *CPAP* variant (c.3947C>A; p. (Thr1316Lys)) in Family 1, which, to our knowledge, has not been previously reported. Given its rarity in population databases, the variant reported here may represent a potential founder mutation in the Pakistani population. Notably, CPAP is highly intolerant to missense (Z score = 1.23) and loss-of-function variants (probability of loss-of-function intolerance [pLI] score = 0.94).

*CPAP* encodes a centrosomal protein that localizes predominantly to spindle poles during pro-metaphase and metaphase. Loss or reduction of CPAP disrupts centrosome integrity, impairs mitotic progression, and interferes with spindle formation (Bond et al., 2005). The variant identified in this study lies within the T-complex protein 10 (TCP) domain at the C-terminus, a region critical for tethering pericentriolar material and mediating protein–protein interactions (Zheng et al., 2014). The C-terminal domain of CPAP also interacts with the microcephaly-associated proteins STIL and CEP135 (Hussain et al., 2012; Kumar et al., 2009), ensuring proper centriole assembly and duplication (Tang et al., 2011; Carvalho-Santos et al., 2012). Importantly, a previously reported missense variant (p.E1235V) within this domain was shown to disrupt STIL binding, thereby impairing centriole biogenesis (Kumar et al., 2009; Tang et al., 2011). By analogy, the p. (Thr1316Lys) variant described here is predicted to induce local misfolding and interfere with CPAP–STIL interactions, ultimately compromising centriole formation.

In Family 2, we identified a novel variant (c.2847 + 5G>T) in *WDR62*, which has not been previously reported in the literature. Interestingly, this variant lies close to the canonical splice donor site and is therefore expected to strongly affect splicing. The NetGene2 server indicated a marked reduction in splice donor probability, from 0.93 (wild type) to 0.38 (mutant), whereas MaxEntScan predicted disruption of the donor site, with the score decreasing from 8.56 to 2.16. Likewise, Mutation Taster predicted a reduced splice donor probability, decreasing from 0.84 (wild type) to 0.47 (mutant). Intriguingly, RNA Splicer predicted that this variant may lead to skipping of exon 23 (128 bp deletion), causing a frameshift and the generation of a premature termination codon (Table 2). *WDR62* is the second most frequently mutated gene associated with primary microcephaly in the Pakistani population, with most reported variants being missense substitutions (Rasool et al., 2020). Functionally, WDR62 is essential for spindle orientation and cortical lamination. In newly formed human neurons, it localizes to nuclear regions, underscoring its critical role in cortical development (Nicholas et al., 2010).

In addition, we identified two families, Families 3 and 4, carrying previously reported and novel variants, respectively, in the *ASPM* gene (HGMD Professional 2025.1, http://www.hgmd.org/) (Khan et al., 2017). Mutations in *ASPM* account for nearly 50% of all reported primary microcephaly cases in the Pakistani population, including 94 nonsense/missense variants, 88 small deletions, 16 small insertions, 2 gross deletions, 18 splice-site changes, and 1 complex rearrangement, totalling 218 pathogenic variants (HGMD Professional 2025.1). The ASPM is essential for the expansion of neural progenitor cells and proper mitotic spindle localization; mutations disrupt symmetric cell division (Fish et al., 2006; Pulvers et al., 2010). Previous studies involving extensive mutational analyses have demonstrated that *ASPM* mutations typically result in protein truncation due to nonsense or frameshift variants, occurring either in compound heterozygous or homozygous states (Fish et al., 2006; Pulvers et al., 2010; Bond et al., 2003). Similarly, the variants identified in this study are predicted to cause loss of function through nonsense-mediated decay. If translation occurs, the resulting truncated proteins lack the C-terminal armadillo-like domain (residues 3,249–3,477) (Nicholas et al., 2009), which mediates interactions with ubiquitin-protein ligase E3A (UBE3A), a centrosomal protein regulating chromosome segregation (Singhmar and Kumar, 2011), and with citron kinase, which controls midbody localization during cytokinesis (Paramasivam et al., 2007). Consequently, these variants are likely to impair protein–protein interactions and subcellular localization of ASPM, resulting in severe functional loss.

This study identifies novel variants in *CPAP, WDR62*, and *ASPM* and further delineates the mutational landscape of microcephaly-associated genes in the Pakistani population. Collectively, these findings expand the spectrum of pathogenic variants linked to primary microcephaly and highlight the importance of genetic counselling for the diagnosis, management, and prevention of this condition.

## Data Availability

The original contributions presented in the study are publicly available. This data can be found in the ClinVar repository with the accession numbers SCV007108600, SCV007108601, SCV007108602, and SCV007108603.
