# Supplementary material for: Expanding the mutational spectrum of congenital microcephaly in Pakistani families
Source: Front Genet. 2026 Jan 5;16:1709083. doi: 10.3389/fgene.2025.1709083 (PMC12812388; doi:10.3389/fgene.2025.1709083)
Supplement: Supplementary file 1 [file DataSheet1.pdf]

Supplementary Material

1 Supplementary Data

Supplementary Material contains two figures and two tables.

2 Supplementary Figures and Tables

2.1 Supplementary Figures

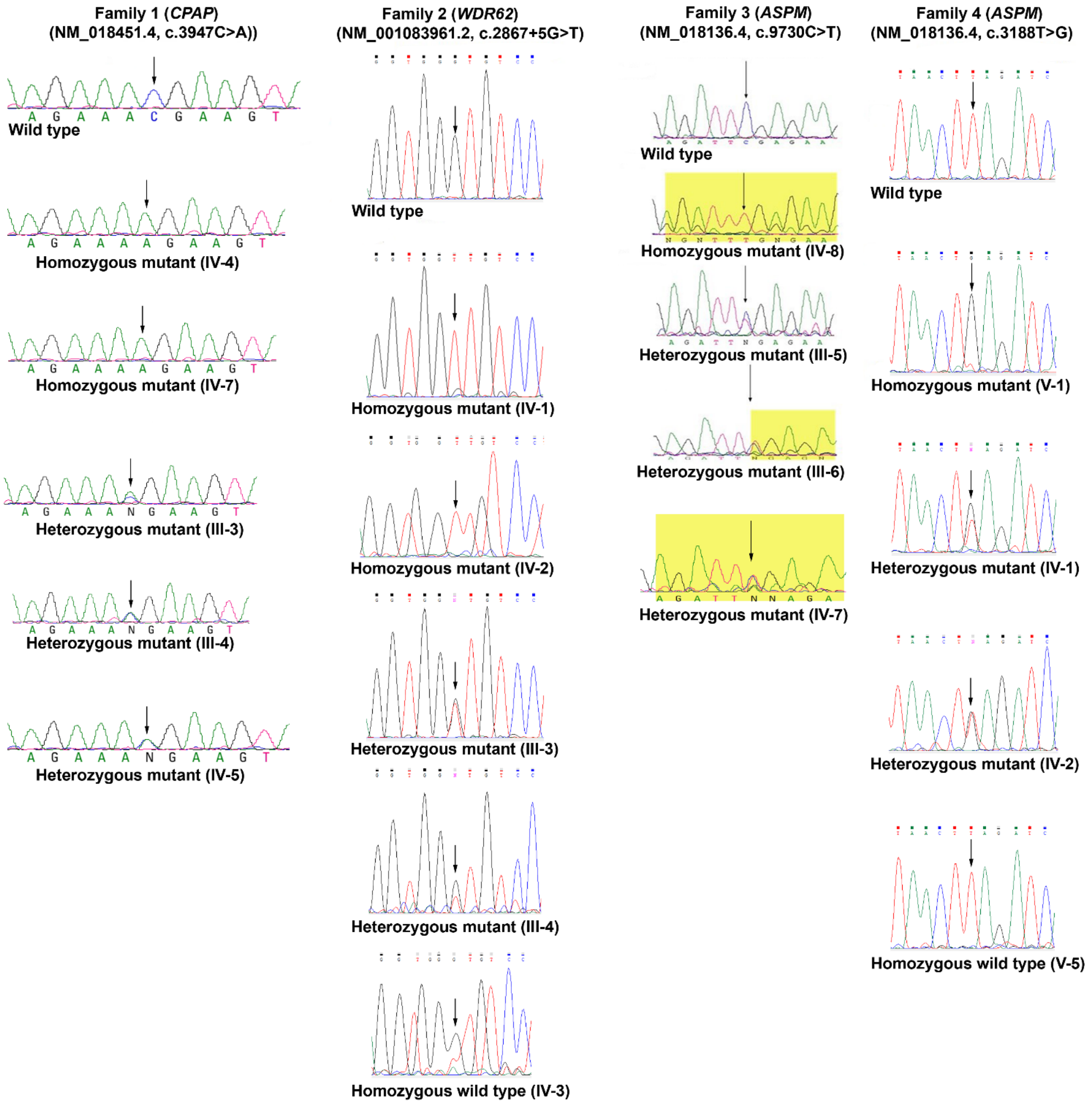

Supplementary Figure 1. Sanger traces showing the co-segregation of *CPAP*, *WDR62*, and *ASPM* variants.

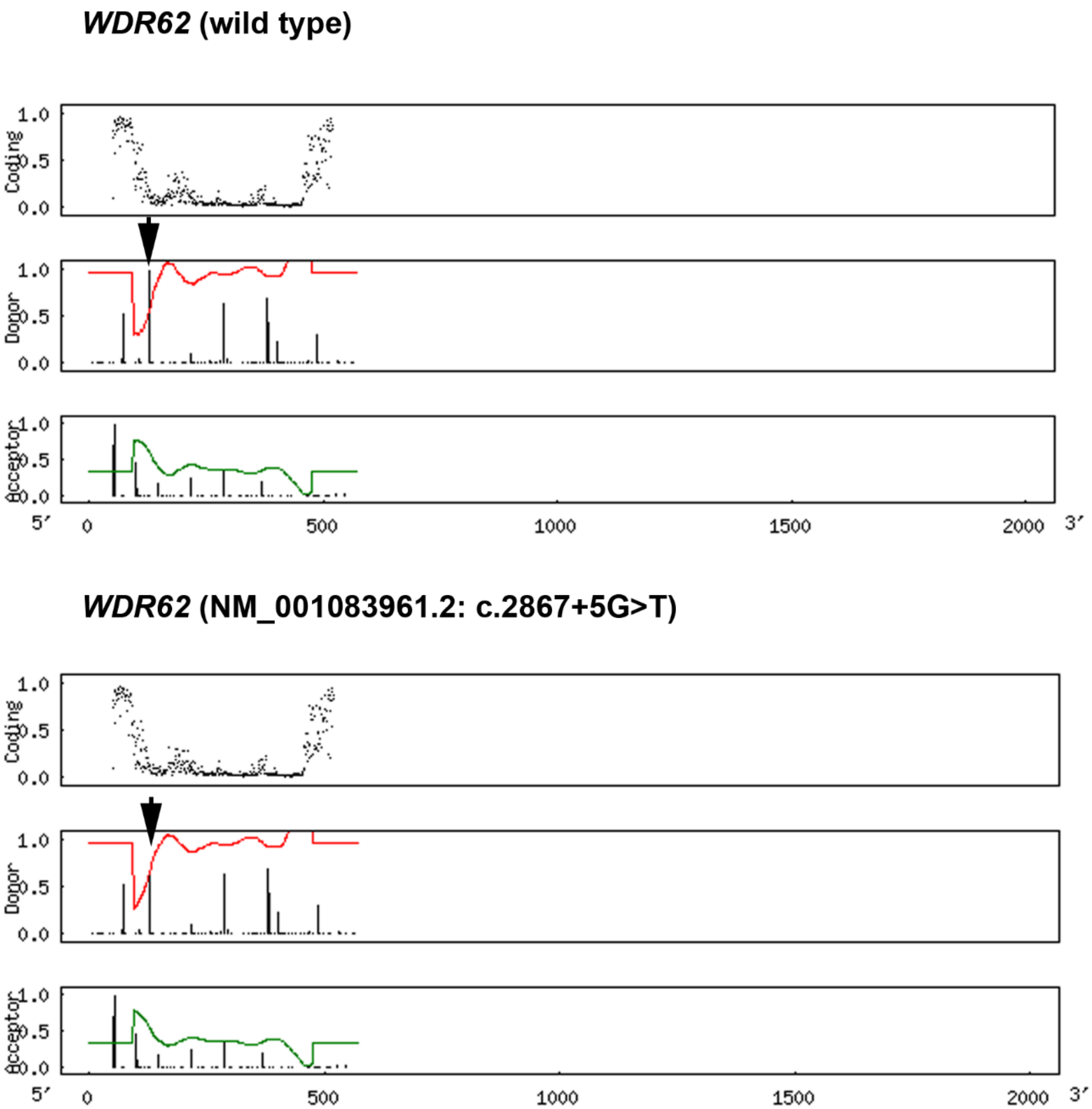

Supplementary Figure 2. The prediction of aberrant splicing by the NetGene2 server. There is a drop in the peak (arrows) at the splice donor site in the mutant compared to the wild type.

2.2 Supplementary Tables

Supplementary Table 1: Oligonucleotide sequences used for the co-segregation analysis

Primer ID	Forward Primer	Reverse Primer
ASPM-Ex13	5'-TCCATTTCAGGCACTTTATTTTC-3'	5'-AAGAAATTCAAGAAAAGACTTCAGG-3'
ASPM-Ex24	5'-GATGTAGATATAAATAGAAAACATTGG-3'	5'-CAGGGGCATATTTGTTGAC-3'
CPAP-Ex17	5'-CAGTGATGGCAACAAACTCATAG-3'	5'-CACAATAAATTAAACAGAATCCACAG-3'
WDR62-Intr23	5'-AGAGAGTCCCCAGGAAGCTG-3'	5'-CCTGTACCCCACCTGGAAC-3'

Supplementary Table 2: Homozygous variants identified in the investigated families by exome sequencing. The causative variants in each family are highlighted in yellow. Allele frequencies shown in columns **P** and **Q** represent data from the in-house CCG database (over 10,000 exomes) and from gnomAD, respectively.
